# Mechanism of Neonatal Intestinal Injury Induced by Hyperoxia Therapy

**DOI:** 10.1155/2022/2316368

**Published:** 2022-01-12

**Authors:** Tian-Ming Li, Dong-Yan Liu

**Affiliations:** Department of Gastroenterology and Medical Research Center, Liaoning Key Laboratory of Research and Application of Animal Models for Environmental and Metabolic Diseases, Shengjing Hospital of China Medical University, Shenyang, Liaoning 110004, China

## Abstract

High concentration oxygen is widely used in the treatment of neonates, which has a significant effect on improving blood oxygen concentration in neonates with respiratory distress. The adverse effects of hyperoxia therapy on the lung, retina, and neurodevelopment of newborns have been extensively studied, but less attention has been paid to intestinal damage caused by hyperoxia therapy. In this review, we focus on the physical, immune, and microorganism barriers of the intestinal tract and discuss neonatal intestinal tract damage caused by hyperoxia therapy and analyze the molecular mechanism of intestinal damage caused by hyperoxia in combination with necrotizing enterocolitis.

## 1. Introduction

High concentration of oxygen is widely used in the treatment of neonates with respiratory distress and has a significant effect on improving their blood oxygen concentration. But oxygen is also a double-edged sword, with toxic effects at high concentrations. The toxicity of oxygen mainly comes from reactive oxygen species (ROS), which include superoxide anions, hydrogen peroxide, nitric oxide, and hydroxyl radicals. Due to its strong oxidizing properties, ROS can react with lipids, proteins, and DNA and damage tissues [1]. Continuous hyperoxia therapy can cause an oversupply of oxygen in the newborn's body. According to reports, membrane-bound NADPH-oxidase (NOX) activation, free radical generation, and DNA damage will occur after inhalation of large amounts of oxygen, leading to cell apoptosis [2]. At present, the damage of hyperoxia therapy to neonatal lung, retina, and nerve development has been confirmed and has become a research hotspot [3–5]. However, there has been less attention paid to intestinal damage caused by hyperoxia therapy. In conclusion, this paper focused on the physical, immune, and biological barriers of the intestinal tract, and combined with necrotizing enterocolitis (NEC) to analyze the molecular mechanism of intestinal damage caused by hyperoxia, and to explore the damage of neonatal intestinal tract caused by hyperoxia treatment.

## 2. Physical Barrier

**Figure 1:**
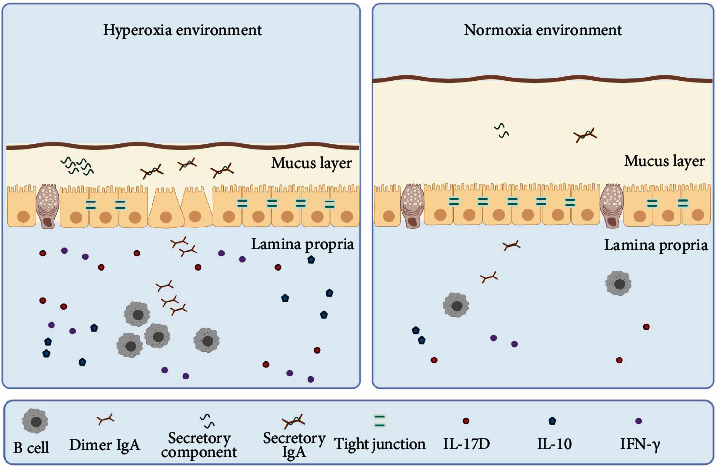
Injury of physical and immune barriers to hyperoxia therapy in neonates. During hyperoxia exposure, the number of goblet cells in the intestine decreased and the mucous layer damaged [10]. Moreover, it inhibits the expression of TJ between cells and increases intestinal permeability [10–12]. In the immune barrier, high oxygen increased SIgA content in the mucus layer by stimulating the SC expression [16, 25]. In addition, high oxygen also increases IL-17D, IL-10, and IFN-*γ*, resulting in a disturbance of cytokine levels [12, 32].

The physical barrier is also called the mechanical barrier. The mechanical barrier consists of the mucus layer on the surface of the intestine, the intestinal epithelial cells and intercellular junctions, and the cells of the lamina propria. The mucus layer is the first line of intestinal innate defense, and mucins in the mucus layer can be divided into gel-forming mucins secreted by goblet cells and transmembrane mucins at the top of intestinal epithelial cells according to their source [6]. There are three connective structures between adjacent intestinal epithelial cells, and the order from apex to basal layer is the tight junction (TJ), adherens junction (AJ), and desmosome. The TJ tightly surrounds the tip of intestinal epithelial cells, which not only blocks harmful molecules but also plays an important role in the absorption of small molecular nutrients through the paracellular pathway [7]. The TJ is composed of two transmembrane protein families, claudin and occludin [8]. On the cytoplasm side, claudin and occludin are connected to the actin cytoskeleton by zonula occludens-1 (ZO-1), and they are important molecules connecting adjacent cells [9]. It has been reported that the intestinal injury score of newborn rats was significantly increased under hyperoxygen stimulation, which was manifested as reduced villus height and crypt depth, intercellular gaps extending to lamina propria, dilated lamina propria capillaries and chylous ducts, and separation of lamina propria from submucosa [10]. Under stimulation by hyperoxia, the number of goblet cells in the intestinal tract of newborn rats was significantly reduced, which can seriously damage the intestinal mucus layer and further aggravate the invasion of bacteria in the intestinal epithelium [10]. The expression of intestinal TJ proteins claudin-4, occludin, and ZO-1 were significantly inhibited by hyperoxygen, resulting in increased intestinal permeability and translocation of bacteria to distant organs (shown in Figure 1) [10–12]. The positive rate of aerobic bacteria in the liver and spleen was 66.7% and 83.3%, respectively, and the positive rate of anaerobic bacteria in both organs was 100% [10]. Following the use of the antioxidant N-acetylcysteine (NAC), the inhibition of high oxygen on the expression of claudin-4, occludin, and ZO-1 in intestinal epithelial cells was significantly restored [12]. It may be due to the breakdown of NAC to produce glutathione, which has a protective effect by removing ROS. Chou et al. treated neonatal rats with cathelicidin in the hyperoxia group and also restored ZO-1 and occludin to control levels [13]. They suggested that the overactivation of NF-*κ*B pathway was responsible for the disruption of the intestinal barrier. NF-*κ*B is an isodimer or heterodimer composed of P65/RelA, RelB, cRel, P50, and P52. In the “resting state,” the dimer of NF-*κ*B binds to I*κ*B in the cytoplasm, and when activated by IKK, the activator of I*κ*B phosphorylates specific serine at the N terminus of I*κ*B protein and released NF-*κ*B into the nucleus to regulate gene expression [14]. The expression levels of RelA and RelB in Caco-2 cells were significantly increased under hyperoxia induction [15]. The level of RelA was also significantly increased in the small intestine of newborn rats at 7 days of age in the hyperoxia group [13]. Low or high doses of cathelicidin restored normal levels of NF-*κ*B in the small intestine and protected the intestinal barrier from hyperoxygen-induced damage [13].

The apoptosis rate and mortality of intestinal epithelial cells were significantly increased under hyperoxia. Zhao's study showed that Caco-2 cells cultured in 85% oxygen for 6 hours had a maximum apoptosis rate of about 15% [16]. The apoptosis rate of the cells decreased gradually with the prolonged incubation time in oxygen [16]. The cell mortality was contrary to the apoptosis rate. With the time of hyperoxia stimulation increasing gradually, the cell mortality was about 30% within 24 hours of culture [16]. Evaluation of apoptosis markers also verified the effect of high oxygen exposure on apoptosis of small intestinal cells, and histone-related DNA fragmentation level in the high oxygen group was significantly higher than that in the control group [17]. During oxidative stress induced by hyperoxygen, the apoptosis signaling pathway of intestinal epithelial cells is significantly activated. Apoptosis signal-regulating kinase 1 (ASK1) is an important member of MAP3K family and can be activated in response to various pressures such as lipopolysaccharide, tumor necrosis factor, and oxidative stress and promote apoptosis through downstream JNK and P38 [18]. With the gradual increase of oxidative stress, the expression of ASK1 in both protein and gene levels gradually increased [15]. In addition, TNF-*α* levels were significantly increased in intestinal lavage fluid of newborn rats and Caco-2 cell line in the hyperoxia group [12, 15]. These results suggest that TNF-*α* may aggravate intestinal barrier injury through apoptosis pathway.

Several tests for markers of intestinal damage have also demonstrated the disruption of the intestinal physical barrier by hyperoxia. Fatty acid binding proteins (FABPs) are soluble small molecular weight proteins in the cytoplasm, which are mainly responsible for the targeted transport of long chain fatty acids in the cell. There are three types of FABPs associated with the gastrointestinal tract, namely, intestinal FABP (I-FABP), liver FABP (L-FABP), and ileal bile acid binding protein (I-BABP) [19]. When intestinal epithelial cells are destroyed, FABP is rapidly released into the blood. Combined with its tissue specificity and short half-life, FABP is an ideal marker for the early diagnosis of intestinal injury [20]. Liu reported that I-FABP and L-FABP in intestinal lavage fluid of newborn rats in the hyperoxia group were significantly higher than those in the control group from the 5th day and continued to increase until the 10th day [12]. Chen's immunohistochemical results also showed that the content of I-FABP in the cytoplasm of small intestinal cells of newborn rats in the 14-day hyperoxia group was significantly higher than that in the control group [11]. Diamine oxidase (DAO) is an important intracellular enzyme that regulates the metabolism of amines, mainly located near the villi tip of the small intestine. DAO level can reflect the integrity and maturity of intestinal mechanical barrier [21]. Liu's study showed that, similar to FABP, DAO level in intestinal lavage fluid of 5-day-old newborn rats was significantly higher than that of the control group and gradually increased with the growth time until day 10 [12]. Lactate dehydrogenase (LDH), which is released outside the cell, is also used to detect cell damage. Li's study showed that blood LDH levels in newborn mice were significantly increased after hyperoxia treatment. Supplementation of arginyl-glutamine or docosahexaenoic acid during the convalescence period after hyperoxia treatment can restore LDH in blood of mice to normal level [17].

## 3. Immune Barrier

The intestinal immune barrier mainly consists of intestinal associated lymphoid tissue, antibodies, and cytokines. Of these, the intestinal related lymphoid tissues are divided into Peyer's patches and solitary lymphoid nodules. In addition, Paneth cells, which are scattered in the intestinal tract, are responsible for secreting lysozyme, M cells, and dendritic cells, and B cells and T cells are responsible for the uptake of antigens.

SIgA plays a central role in intestinal mucosal immunity. SIgA is composed of a J chain, SC, and two IgA monomers, which are linked to form dimer IgA (DigA) by the cysteine residue of the Fc segment in the J chain [22]. DigA is secreted by plasma cells near the surface of the intestinal tract. During transport to the lumen, DigA binds to the polymerized immunoglobulin receptor (PIGR) on the base of intestinal epithelial cells and enters these cells by endocytosis [23]. At the apex of the intestinal epithelial cells, protease cleaves the extracellular portion of the PIGR to form the SC, which forms a complex with DigA and is released into the mucosa. This SC protects SIgA from degradation. In addition, 50% of the PIGR in the human body is transferred to the top surface without DigA and is released as free SC after being cut [24]. Free SCs can remove pathogenic bacteria and bacterial toxins in the form of protein-protein or protein-glycan interactions [24]. Studies have shown that intestinal epithelial cells express increased SCs and promote intestinal SIgA production under hyperoxia stimulation [16, 25]. High levels of SCs and SIgA will help maintain intestinal homeostasis and have a positive protective effect on neonates exposed to hyperoxia.

Interleukin (IL)-17D, a new member of the IL-17 family, is of great concern in the case of intestinal damage caused by high oxygen levels. IL-17D is composed of 202 amino acids, and it has 25% homology with IL-17A, a prototype member of the IL-17 family [26]. The primary function of the IL-17 family is to indirectly regulate the immune response by regulating the expression of cytokines. IL-17D can stimulate the expression of IL-6, IL-8, and granulocyte macrophage colony-stimulating factor (GM-CSF) in endothelial cells, which is similar to other members of the family [26, 27]. IL-17D is expressed in multiple organs and tissues throughout the body, such as the heart, lung, brain, pancreas, skeletal muscle, and adipose tissue. It is also widely involved in infections, tumors, and autoimmune diseases [26]. Mice deficient in IL-17D not only showed stronger tumor susceptibility but also showed more severe infection symptoms after infection with vaccinia virus (VV) and murine cytomegalovirus (MCMV), such as longer scar length and lower body weight [28]. In addition, IL-17D can stimulate tumor endothelial cells to express monocyte chemotactic protein 1 (MCP-1) and recruit NK cells and macrophages into the tumor microenvironment to play an antitumor role [29]. These studies all suggest that IL-17D plays a positive role in protecting the host against tumor and infection. However, reports of sepsis and listeria infections have also revealed the harmful side of IL-17D. The levels of IL-17D were significantly elevated in patients with sepsis. IL-17D inhibits the phagocytosis of macrophages by inhibiting activation of the nuclear factor- (NF-) *κ*B signaling pathway and ultimately aggravates the symptoms in sepsis patients [30]. Lee et al. found that IL-17D-deficient mice infected with listeria exhibited greater CD8 + T cell activity. Further studies have confirmed that IL-17D inhibits CD8 + T cell activation by inhibiting dendritic cell activation [31]. In conclusion, IL-17D plays a comprehensive regulatory role in anti-infection and antitumor by relying on immune cells, rather than simply promoting or inhibiting the development of disease. IL-17D is expressed by CD4 + T cells, CD19 + B cells, and villous epithelial cells in the small intestine. Under stimulation by hyperoxia, the expression of IL-17D in CD4 + T cells and CD19 + B cells gradually increased with prolongation of hyperoxia exposure, while the expression of IL-17D in intestinal epithelial cells reached a peak at 7 days and then gradually decreased [32]. Nrf2 is an important transcription factor regulating the IL-17D expression, and Nrf2 is inhibited by Keap1 in the cytoplasm under the resting state. Nrf2 is released during oxidative stress and activates the transcription of cell protective genes [33]. Liu et al. found that Nrf2 and IL-17D expression increased significantly under hyperoxia conditions. Furthermore, the expression of IL-17D was further increased after Nrf2 was activated with tert-butylhydroquinone (TBHQ) [34]. This suggests that during hyperoxia, intestinal epithelial cells and immune cells respond to the damage caused by hyperoxia by promoting the inflammatory response through IL-17D (shown in Figure 1).

In addition, intestinal IFN-*γ* and IL-10 levels were significantly increased under high oxygen conditions [12]. IFN-*γ* and IL-10 are closely related, and they both belong to the second class cytokine family. Moreover, all of their receptors are dimer receptors and contain IL-10R*β* [35]. However, IFN-*γ* and IL-10 have very different functions. IFN-*γ* is a proinflammatory factor that reduces barrier integrity and leads to bacterial and immune cell translocation [36]. IL-10 is an anti-inflammatory cytokine that inhibits the expression of tumor necrosis factor- (TNF-) *α* and thus protects the body from hyperoxia-induced inflammatory damage. Both IFN-*γ* and IL-10 are elevated, suggesting that disruptions in cytokine levels may cause intestinal damage (shown in Figure 1).

## 4. Intestinal Flora and the Gut-Brain/Lung Axis under Hyperoxia

The intestinal flora of the newborn is easily affected by the external environment. Dysbiosis in the gut affects the development of distant lungs and the brain, these mechanisms are known as the “gut-lung axis” and “gut-brain axis,” and this adverse effect can persist even into adulthood [37]. With regard to the “gut-brain axis,” animal models have demonstrated that intestinal bacteria and their metabolites can affect central nervous system homeostasis and thus alter animal behavior [38]. Lo et al. reported that intestinal bacterial diversity decreased in mice 7 days after exposure to high oxygen [39]. Proteus was 98.7% in the intestinal tract of mice in the hyperoxia group, while proteus was about 80%, and Firmicutes were 19.6% in the intestinal tract of mice in the air group [39]. After 42 days of birth, the proportion of intestinal microbes in the hyperoxia group and the air group remained significantly different: The hyperoxia group was dominated by Epsilonbacteraeota (53.1%), while the air group was dominated by Firmicutes (61.8%) [39]. In addition, 42 days after birth, not only myelin formation was damaged but also the number of apoptotic cells in brain was significantly higher in the hyperoxia group than in the air group [39]. Abnormalities in brain development led to reduced social skills and motor coordination in these oxygen-exposed mice during adolescence [39]. Combined with these findings, Lo suggested that changes in the intestinal flora of newborn mice exposed to high oxygen could affect brain development and thus alter adolescent behavior [39].

The “gut-lung axis” study uses “cytokines” to link intestinal microbiota disorders to lung disease. In a model of postpartum growth restriction, hyperoxia leads to an increase in Gram-negative enterobacteria in the distal small intestine [40]. Activation of TLR4 by Enterobacteriaceae increases circulating levels of IL-1*β*, which transmits inflammatory signals to the lungs [40]. THE I*κ*B expression in the lungs is significantly reduced, aggravating NF-*κ*B-mediated pulmonary inflammatory response, and ultimately leading to pulmonary hypertension and right ventricular hypertrophy [41]. Treatment with the TLR4 inhibitor TAK-242 not only reduced circulating IL-1*β* levels but also significantly increased pulmonary I*κ*B*α* levels and reduced pulmonary hypertension levels in the pups of the growth restriction model [41].

In the antibiotic exposure model, it was also found that hyperoxia led to an increase in the Bacteroidales and Alistipes in the intestinal tract of neonates, and it was also found that the abundance of Akkermansia was significantly reduced [42]. Decreased levels of Akkermansia, a probiotic, have been linked to the development of obesity, type II diabetes, inflammatory bowel disease, and the childhood allergy atopy [43, 44]. Studies have shown that Akkermansia can stimulate the expression of IL-8 in intestinal epithelial cells, and this low level of proinflammatory stimulation may keep the mucosal-related immune system at an appropriate level, which plays an important role in maintaining the integrity of intestinal epithelial cells [45].

## 5. Hyperoxia and Necrotizing Enterocolitis

NEC is the most common and fatal gastrointestinal disease in newborns, which can rapidly progress to systemic sepsis and lead to infant death if left untreated. The etiology of NEC is complex and diverse and is currently believed to be due to intestinal tissue necrosis caused by preterm birth, formula feeding, oxidative stress, and pathogenic bacteria colonization [46].

TLR4 is one of the most important pathways in the pathogenesis of NEC. The expression level of intestinal TLR4 was significantly increased in both a hyperoxia model and NEC model [10]. Chou reported that the expression of TLR4 in intestinal epithelial cells of newborn rats increased significantly after 7 days of hyperoxia exposure [10]. The repair of intestinal injury mainly involves two aspects: healthy intestinal cells migrate laterally to the damaged area to repair the intestinal barrier or immature intestinal epithelial cell precursors differentiate and mature to replace the dead cells. Activation of TLR4 not only inhibits the migration of intestinal cells but also disrupts the differentiation of intestinal stem cells into goblet cells through Notch signaling [47]. TLR4 knockout mice showed higher goblet cell levels and reduced NEC severity [48, 49]. The addition of probiotics to formula milk can significantly reduce the expression of TLRs by intestinal cells, thereby reducing the inflammatory response of the intestinal tract, but high oxygen exposure weakens this protective effect [50].

Intracellular ROS come from mitochondria and the NADPH enzyme family in the cytoplasm, of which the mitochondrial ROS account for the majority. Oxidative damage to mitochondria caused by hyperoxia can lead to significant accumulation of ROS, which will further damage mitochondrial DNA and eventually lead to cell death [51]. Studies have shown that H_2_O_2_ can promote the activation of IKK and then activate NF-*κ*B. The use of tuna skeleton protein (APTBP) reduced the protein level of H_2_O_2_-activated IKK, improved the NEC phenotype and reduced the inflammatory response [52]. In addition, the use of TNF-*α* antagonist or TNF-*α* receptor antibody in the NEC model significantly reduced the intestinal oxidative stress response and intestinal tissue damage in NEC [53, 54].

Finally, the use of antioxidants to treat NEC has been widely validated. Using antioxidants in vivo such as NAC, Ginger (Zingiber officinale Roscoe), Nigella sativa oil (NSO), and caffeic acid phenethyl ester (CAPE) is effective in the treatment of NEC [55–58].

## 6. Conclusion

In this article, we systematically reviewed the mechanisms of neonatal intestinal damage caused by hyperoxia and summarized the role of hyperoxia in NEC. In order to effectively prevent intestinal damage caused by hyperoxia therapy, we should conduct a more comprehensive study on the molecular mechanisms of oxidative stress and inflammation caused by hyperoxia therapy. Finally, neonates are at a critical stage of development, and hyperoxia therapy may adversely affect the immune and digestive functions of the neonates' intestines, even into adolescence. This suggests that doctors should be more cautious about the use of hyperoxia in clinical practice and pay more attention to the digestive system during patient follow-up.

## Data Availability

The data and code generated or analyzed in this study are available from the corresponding author upon reasonable request.

## Additional Points

*Core Tip*. In this review, we comprehensively review the mechanisms by which hyperoxia therapy damages the intestinal tract of newborns. We divided the intestinal injury into three parts: physical barrier, immune barrier, and microorganism barrier and analyzed the regulation of intestinal cell signaling pathway by hyperoxia combined with necrotizing enterocolitis. We hope that through this review, people pay more attention to the damage of neonatal intestinal tract caused by hyperoxia and use hyperoxia more carefully to avoid the harm of hyperoxia.
